# The Role of Configurational Entropy in Amorphous Systems

**DOI:** 10.3390/pharmaceutics2020224

**Published:** 2010-05-25

**Authors:** Kirsten A. Graeser, James E. Patterson, J. Axel Zeitler, Thomas Rades

**Affiliations:** 1 School of Pharmacy, University of Otago, Dunedin, New Zealand; 2 Pharmaceutical Development, GlaxoSmithKline, Harlow, CM19 5AW, UK; 3 Department of Chemical Engineering and Biotechnology, University of Cambridge, Pembroke Street, Cambridge CB2 3RA, UK

**Keywords:** configurational entropy, amorphous, molecular mobility, solubility

## Abstract

Configurational entropy is an important parameter in amorphous systems. It is involved in the thermodynamic considerations, plays an important role in the molecular mobility calculations through its appearance in the Adam-Gibbs equation and provides information on the solubility increase of an amorphous form compared to its crystalline counterpart. This paper presents a calorimetric method which enables the scientist to quickly determine the values for the configurational entropy at any temperature and obtain the maximum of information from these measurements.

## 1. Introduction

In recent years the amorphous state has gained increasing interest in pharmaceutics due to its favourable properties compared to its crystalline counterpart [1,2]. These advantages include increased solubility and potential higher bioavailability, making the amorphous state a promising approach for delivering poorly soluble drugs.

However, major issues when dealing with the amorphous state are physical and chemical instabilities, and to date the prediction of physical and chemical stability of drugs in the amorphous state still proves challenging. Often it is observed that the amorphous state of a compound that had been prepared by different methods, shows differing physico-chemical properties and stability [3]. In order to increase the stability of the amorphous state of a drug, these are often formulated with a hydrophilic polymer using a variety of preparation techniques, creating a solid dispersion or glass solution [4]. In a glass solution the drug is molecularly dispersed in a polymer matrix.

**Figure 1:**
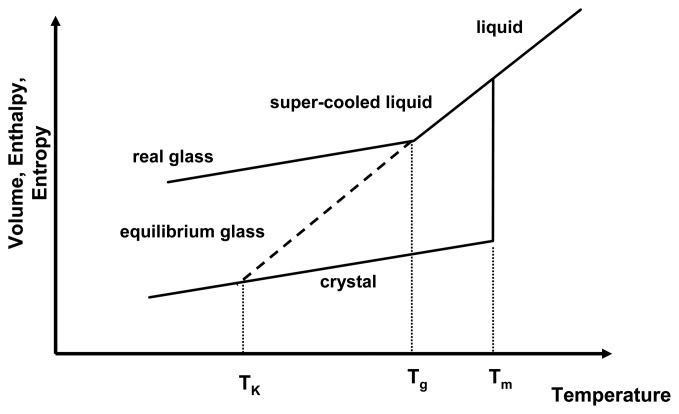
Thermodynamic relationship of crystalline and amorphous state as a function of temperature. Shown are the melting temperature (*T_m_*), the glass transition temperature (*T_g_*) and the Kauzmann temperature (*T_K_*). Adapted from [1].

Compared to the crystalline form of a drug, the amorphous form is in a state of higher energy. This is due to the fact that the amorphous state possesses excess thermodynamic properties such as enthalpy, entropy and Gibbs free energy. Figure 1 depicts the relationship of the thermodynamic properties and temperature for the amorphous and crystalline state. As a liquid melt of a crystal is cooled rapidly, recrystallisation may be prevented and the slope of the equilibrium liquid line may be followed below the melting temperature, *T_m_*, resulting in a gradual decrease of thermodynamic properties below *T_m_*. This is the super-cooled liquid state, in which viscosity is low (typically around 10^−3^ − 10^12^ Pa s) and mobility of the molecules is high and they are able to follow any further decrease of temperature to attain equilibrium conditions. However, upon further cooling, at the glass transition temperature, *T_g_*, the molecules cannot follow the decrease in temperature any longer and the systems solidifies, falling out of equilibrium. This is represented by the change of the slope in Figure 1. Below the *T_g_*, the system is in the glassy state, exhibiting high viscosities of > 10^12^ Pa s.

If the *T_g_* did not occur and the system remained in equilibrium throughout the cooling process, the super-cooled liquid line would be followed (now as an equilibrium glass) and it would intersect the crystal line at the Kauzmann temperature, *T_K_*. Below the *T_K_*, the amorphous system would have lower entropy and enthalpy than the crystalline state, which presents a violation of the laws of thermodynamics. Therefore, the occurrence of the *T_g_* has two implications for amorphous systems: firstly glasses are in a non-equilibrium state and equilibrium thermodynamics cannot be applied below that temperature and secondly the physico-chemical properties of amorphous systems above and below the *T_g_* are different.

During storage, it is often observed that the amorphous state reduces its excess enthalpy and entropy without recrystallising. This is called “relaxation” as the amorphous state relaxes towards a lower energetic state, still remaining in the amorphous form. The real glass relaxes asymptotically towards the equilibrium glassy state. This relaxation behaviour can be seen as an indication that below the *T_g_* mobility is low, but still existent.

The higher energetic state is beneficial in terms of improving the solubility of a compound, however, it is detrimental to the physical stability [5,6]. Due to the enhanced thermodynamic properties of the amorphous state compared to the crystalline state, recrystallisation provides a means of reducing this excess free energy but in doing so, the solubility advantages are negated.

Crystallisation is a result of a nucleation process, where stable nuclei are created, followed by crystal growth. The recrystallisation of an amorphous form is governed by the same factors as the crystallisation from a melt [1], and therefore the crystallisation processes have been described on the basis of the classical nucleation theory (CNT) for homogenous nucleation [7].

Nucleation is a process which involves the overcoming of a potential barrier and was first described by Gibbs [8]. The change of Gibbs free energy (Δ*G*), due to the formation of a cluster of the new phase (crystal) is given by Equation 1:

(1) $$\Delta G=\Delta G_{V}+\Delta G_{S}$$

where Δ*G_S_* is the change in surface free energy (in J mol^−1^) and Δ*G_V_* is the volume free energy change (in J mol^−1^).

The value of Δ*G_S_* is associated with the formation of the cluster and represents a positive quantity. The value of Δ*G_V_* is associated with the phase transition of liquid to solid and represents a negative quantity [9]. According to the CNT, when nucleation occurs, clusters grow and decay until a stable nucleus is formed. After a cluster of a critical size has been formed, it will grow in size and hence recrystallisation of the amorphous form takes place.

Nucleation and crystal growth are not solely governed by thermodynamics but also require the individual molecules to move via diffusion. This kinetic proportion of the recrystallisation effect is usually considered as the molecular mobility or its reciprocal, the relaxation time *τ*. It has long been suggested that molecules exhibit sufficient mobility only at temperatures close to or above the *T_g_*, and storage at temperatures below *T_g_* would ensure physical stability. It has however been found that nucleation and crystal growth also occur at temperatures well below the *T_g_*, however, the relative rate may be much slower than in the temperature region above the *T_g_* [10,11].

Recrystallisation should therefore be influenced by thermodynamic (such as enthalpy, entropy and Gibbs free energy) and kinetic parameters (such as mobility). A commonality of the thermodynamic and kinetic parameters is the involvement of the configurational entropy, *S*_conf_, which is the difference in entropy between the amorphous and the crystalline state.

### 1.1. Thermodynamic involvement of S_conf_

The desired properties of the amorphous state (higher solubility and dissolution rate compared to the crystalline form) have been attributed, at least in part, to an increase in thermodynamic properties, e.g., free energy, entropy and enthalpy. This change in free energy is regarded as a driving factor for recrystallisation: the larger the difference in free energy between the amorphous and crystalline state the more thermodynamically favorable the situation will be upon recrystallisation.

The difference in Gibbs free energy between the amorphous and the crystalline states can be calculated using enthalpic and entropic values for the amorphous and crystalline state as shown in Equation 2:

(2) $$G_{\text{conf}}=H_{\text{conf}}(T)+S_{\text{conf}}(T)$$

The term “configurational” denotes the difference between the amorphous and the crystalline state and the parameters *H*_conf_ and *S*_conf_ may be calculated from their relationship with the heat capacity.

(3) $$H_{\text{conf}}(T)=H^{\text{amorph}}(T)-H^{\text{crystal}}(T)$$

(4) $$S_{\text{conf}}(T)=S^{\text{amorph}}(T)-S^{\text{crystal}}(T)$$

(5) $$H_{\text{conf}}=\Delta H_{m}+\int_{T_{m}}^{T}Cp_{\text{conf}}dT$$

(6) $$S_{\text{conf}}=\Delta S_{m}+\int_{T_{m}}^{T}\frac{Cp_{\text{conf}}}{T}dT$$

where *H*_conf_ is the configurational enthalpy (in J mol^−1^), *S*_conf_ the configurational entropy (in J mol^−1^ K^−1^), Δ*H_m_* the melting enthalpy of the crystal (in J mol^−1^) and Δ*S_m_* the melting entropy of the crystal (in J mol^−1^ K^−1^).

The melting entropy can be obtained from the following relationship:

(7) $$\Delta S_{m}=\frac{\Delta H_{m}}{T_{m}}$$

The configurational thermodynamic values give an indication of the relationship between the amorphous state and the crystalline state of a compound. The larger the configurational values are the greater are the differences between the crystalline and the amorphous states.

**Figure 2:**
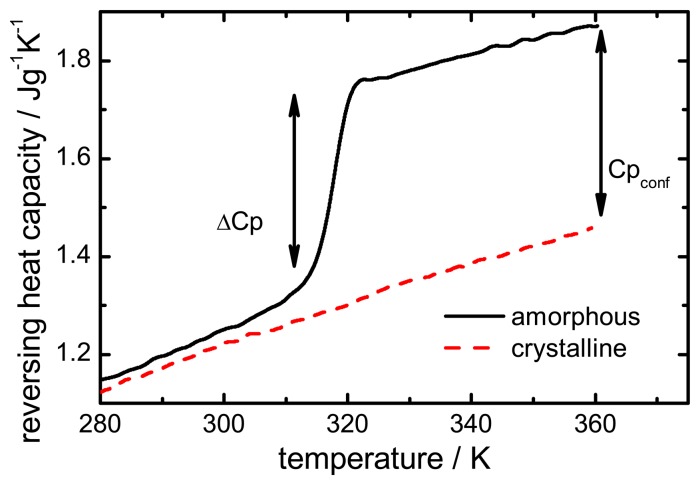
Heat capacity difference (ΔCp) and configurational heat capacity (Cp**_c_**_onf_) of indomethacin.

The configurational heat capacity *Cp*_conf_, required to calculate the configurational properties, is the difference between the amorphous and the crystalline heat capacities. *Cp*_conf_ is not identical to Δ*Cp*, which denotes the heat capacity change of the amorphous state at *T_g_* as shown in Figure 2.

Below the *T_g_* the heat capacity values of the glass and the crystal may be similar, however, the heat capacities are not identical, therefore, *Cp*_conf_ is never zero.

After passing through the *T_g_* the *Cp*_conf_ of an amorphous compound may increase or decrease with temperature or follow a specific temperature dependence, depending on the properties of the material [12,13]. The temperature dependence of the configurational heat capacity above *T_g_* has been described by the hyperbolic relation presented in the following equation:

(8) $$Cp_{\text{conf}}=\frac{K}{T}=\frac{Cp_{\text{conf}}(T_{g})T}{T}$$

### 1.2. Kinetic involvement of S_conf_

The amorphous state not only possesses higher thermodynamic properties compared to the crystalline state, it also shows enhanced molecular mobility [5,14] and this is considered an important factor for the subsequent physical and chemical instabilities [1,15]. Crystal nuclei formation, the first step of the recrystallisation process, is a result of the localized faster mobility of molecules [16] and degradation reactions such as hydrolysis or protein degradation have been attributed to increased molecular mobility within the amorphous state [17].

Molecular mobility has therefore been the topic of numerous investigations, however, due to the complex nature of the amorphous state and the still poorly understood relaxation properties, to date no single equation can be used to estimate the relaxation time of the amorphous state. Among those generally used are the Kohlrausch-Williams-Watts equation (KWW) [18], the Adam-Gibbs equation (AG) [19] and the Vogel–Tamman–Fulcher equation (VTF) [20,21,22].

These equations all show advantages and disadvantages as they address different issues, but the most commonly used equation to estimate the molecular mobility for the temperature range below the *T_g_* is the AG.

The governing thought of the AG equation is that a liquid consists of regions that rearrange themselves in units, the so-called cooperatively rearranging regions (CRR). Upon cooling from the super-cooled liquid state, these CRR become progressively larger. The size of the CRR is determined by the difference in the configurational entropy, *S*_conf_, of the liquid which varies with temperature. When the temperature is high, the Sconf is large and the size of the CRR is small. Upon cooling, the *S*_conf_ decreases and in return the size of the CRR increases. In the AG theory, this increasing cooperativity is believed to be due to a loss of configurational entropy, which enables the calculation of molecular mobility [5].

(9) $$\tau (T)=\tau_{0}\exp\left(\frac{\Delta \mu s_{\text{conf}}^{*}}{k_{B}TS_{\text{conf}}(T)}\right)$$

with *τ* relaxation time below *T_g_* (in s), *τ*_0_ pre-exponential parameter (lifetime of the atomic vibrations, 10-14 s), Δ*μ* activation energy of cooperative rearrangement (J mol^−1^), $s_{\text{conf}}^{*}$ entropy of smallest cooperative molecular region (J mol^−1^ K^−1^), *k_B_* Boltzmann constant (1.38 J K^−1^) and *S*_conf_(*T*) configurational entropy at temperature T (J mol^−1^ K^−1^).

Equation 9 simplifies to the following expression if the properties of the glass forming liquid (Δ*μ* and $s_{\text{conf}}^{*}$) are considered constant:

(10) $$\tau (T)=\tau_{0}\exp\left(\frac{C}{TS_{\text{conf}}(T)}\right)$$

where *C* is a constant.

**Figure 3:**
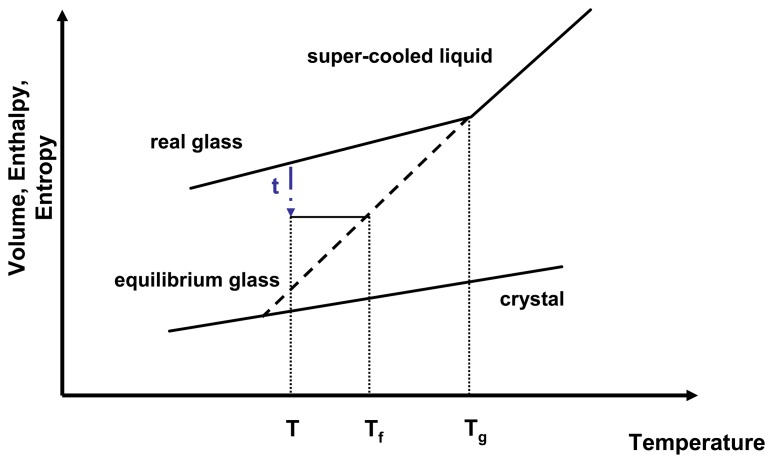
Relationship of enthalpy and temperature for an amorphous system. *τ* represents the relaxation time, *T* is the isothermal relaxation temperature, *T_f_* is the fictive temperature and *T_g_* is the glass transition temperature. Adapted from [24].

By applying Equation 10 it has to be considered that the entropic contributions are entirely due to *S*_conf_ and any other influence (e.g., vibrational entropy) is neglected [23]. As a result, *S*_conf_ has a direct influence on the relaxation time and therefore on molecular mobility. As a glass relaxes isothermally, it reduces a portion of its excess entropy which in return leads to an increase of relaxation time. The molecular mobility of an amorphous state therefore is not only temperature dependent but also time dependent. A convenient way to express the temperature and time dependence of the molecular mobility is to introduce the fictive temperature, *T_f_* (Figure 3).

A real glass is not in equilibrium, therefore applying thermodynamic principles proves challenging. However, thermodynamics can be estimated by relating the real glass to the theoretical equilibrium glass at the same temperature.

The fictive temperature is defined as the temperature at which the system under investigation has the same thermodynamic properties as its equilibrium state at that temperature and that time. The configurational entropy can therefore be described by Equation 12.

(11) $$S_{\text{conf}}^{g}(T)=S_{\text{conf}}^{e}(T_{f})=\int_{T_{0}}^{T_{f}}\frac{Cp_{\text{conf}}}{T}dt$$

with $S_{\text{conf}}^{g}$ configurational entropy of the real glass (in J mol^−1^ K^−1^), $S_{\text{conf}}^{g}$ configurational entropy of the equilibrium super-cooled liquid (in J mol^−1^ K^−1^) and *T_f_* fictive temperature (in K).

The fictive temperature is a convenient way of describing the temperature and time dependence of *S*_conf_ for real glasses and enables calculation of molecular mobility in these systems.

Through a number of rearrangements and substitutions of *S*_conf_ in Equation 10 [15] the AG equation can be written as:

(12) $$\tau (T)=\tau_{0}\exp\left(\frac{DT_{0}}{T-(T/T_{f})T_{0}}\right)$$

where *D* is the dimensionless Angell's strength parameter and *T*_0_ is the temperature of zero *S*_conf_ (in K).

The relaxation time *τ* may therefore be expressed by the fictive temperature which contains the contributions from the configurational entropy.

### 1.3. Involvement of S_conf_ in solubility prediction

Amorphous compounds show a higher apparent solubility than their crystalline counterparts [25] due to their higher energetic state and the disordered structure that does not require the crystal lattice to be broken upon dissolution. According to studies by Parks et al. the theoretical solubility ratio (*σ*^amorph^*/σ*^crystal^) of the amorphous and crystalline form at a given temperature can be described through the free energy difference between the two forms [26,27]:

(13) $$G_{\text{conf}}=RT\;\ln\left(\frac{\sigma^{\text{amorph}}}{\sigma^{\text{crystalline}}}\right)$$

with the gas constant *R* = 8.314 J mol^−1^ K^−1^.

The value of *G*_conf_ can be calculated as presented in Equation 2, via *Cp*_conf_ and hence *S*_conf_. This approach has already been successfully applied for estimating the solubility differences between different crystalline polymorphs [28,29].

In the literature experimental solubility increases of up to 10 fold have been reported [30] for amorphous systems, which in return can increase the bioavailability of a poorly soluble compound considerably. However, for amorphous compounds the calculated solubility may increase by up to 1,600 fold [30,31]. However, this potential increase usually is not observed in vitro and the estimated values are significantly smaller. This is partly due to the fact that the amorphous state is far from equilibrium which poses difficulties in determining their equilibrium thermodynamic properties and partly due to the tendency of the amorphous state to revert back to the crystalline state upon exposure to solvents such as water or biorelevant media [30]. It is also observed that the amorphous state does not recrystallise to its original crystalline state but may crystallise to a different, potentially less soluble, polymorph [32]. This is not trivial and can pose challenges during development of drug formulations.

Despite their inaccurate values for absolute solubility, the estimated solubility ratios are a useful tool for estimating the theoretical maximal solubility and serve as an indication of the theoretical driving force for dissolution.

These considerations regarding the *S*_conf_ highlight the importance of this parameter in terms of amorphous stability and solubility, the major factors of interest in pharmaceutics.

This paper will focus on the calculation of *S*_conf_ from simple calorimetric measurements and will give details of the methods used. It goes on to calculate the important thermodynamic, kinetic and solubility parameters. It is not in the scope of this article to compare calculated values to experimentally determined values or provide in depth interpretation of results.

## 2. Results and Discussion

### 2.1. Thermodynamic involvement of S_conf_

**Figure 4:**
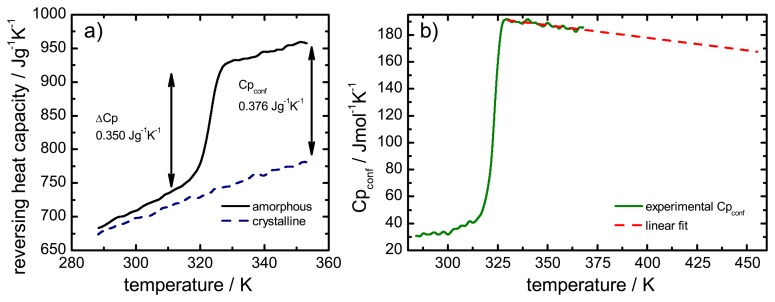
a) Heat capacity of the crystalline (dashed line) and amorphous (solid line) form and configurational heat capacity of lacidipine. Δ*Cp* is the heat capacity change at the *T_g_*, *Cp*_conf_ is the difference between the heat capacities of the amorphous and crystalline forms. b) Experimental *Cp*_conf_ from a) (solid line) and extrapolated *Cp*_conf_ (dashed line) for the temperature range above *T_g_* to *T_m_*.

The configurational heat capacities for all drugs were calculated from the individually measured heat capacities of the glass, the liquid and the crystalline form. Figure 4a presents the results of the heat capacities of lacidipine measured in a temperature interval about 50 K above and below the *T_g_*. Subtraction of the crystalline values from the amorphous values (above and below *T_g_*) resulted in the graph presented in Figure 4b. The obtained values above *T_g_* were extrapolated to the melting temperature.

**Figure 5:**
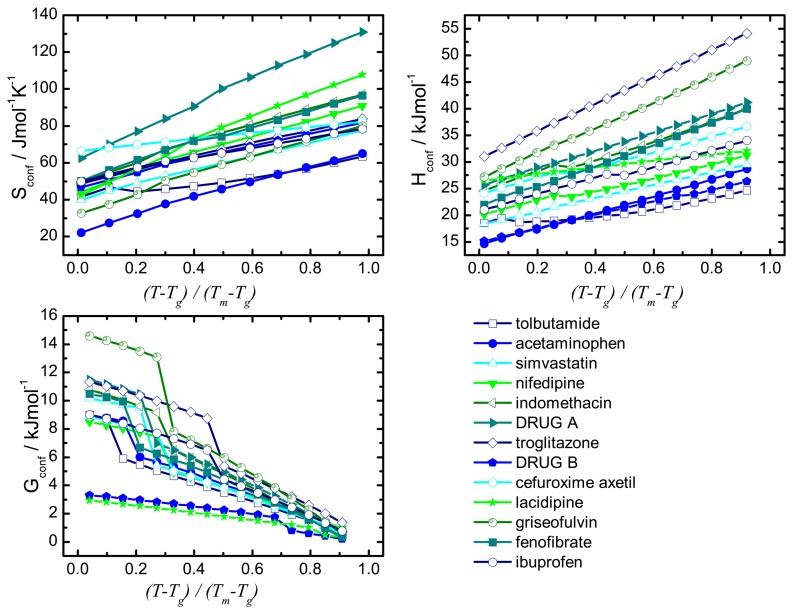
Temperature dependence of the configurational thermodynamic properties.

The configurational thermodynamic parameters were calculated from the extrapolated *Cp*_conf_ values and their respective temperature evolution is presented in Figure 5. For ease of comparison the results are presented on a normalised temperature scale, which allows a direct comparison of drugs with different glass transition and melting temperatures [33].

The temperature range plotted covers the midpoint of the glass transition temperature (0.0) to the melting temperature (1.0). Increasing the temperature of the system resulted in an increased thermal energy within the system which in return increased the entropy for the super-cooled liquid and the crystal. An overall configurational entropy increase was observed as the entropy of the amorphous state (here: super-cooled liquid) was higher and exhibited a stronger temperature dependence than the entropy of the respective crystal. Through extrapolation of *Cp*_conf_, the value for the heat capacity difference at any temperature may be determined and hence the *Sp*_conf_.

**Figure 6:**
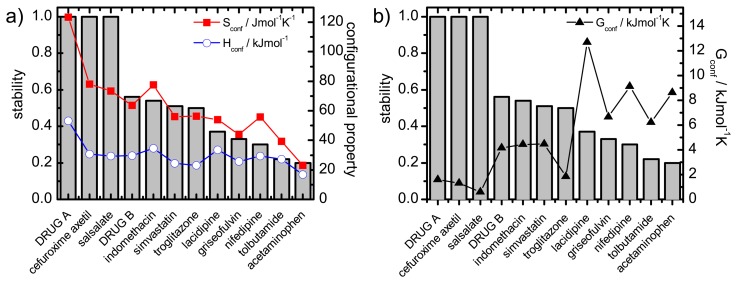
(a) Correlation of configurational entropy and enthalpy (b) Correlation of configurational Gibbs free energy with stability above the *T_g_* [34].

Stability studies above *T_g_* were conducted to relate the thermodynamic parameters with stability (Figure 6) and details of this procedure can be found in [34].

Linear regression of the thermodynamic parameters with stability showed the *S*_conf_ to exhibit the largest degree of correlation, suggesting that above the *T_g_*, the *S*_conf_ had the greatest influence on stability (Table 1).

**Table 1:** Regression coefficients of correlating physical stability above *T_g_* with thermodynamic parameters.

	*S*_conf_	*H*_conf_	*G*_conf_
*r*^2^	0.679	0.336	0.628

### 2.2. Kinetic involvement of S_conf_

Due to the time dependence as well as the temperature dependence of the configurational entropy in a real glass (not in equilibrium), the values for *S*_conf_ that were calculated previously cannot be entered directly into the AG equation. It was already mentioned that *S*_conf_ appears in the AG equation in form of the fictive temperature, *T_f_*. The required parameters to calculate the relaxation time via the AG equation were calculated by following the procedure outlined in the Experimental Section. The results for the individual parameters are presented in Table 2.

It can be seen that the relaxation time (and hence the mobility) was different for the sample set of drugs. The values shown here were calculated at a temperature 20 K below the glass transition of the individual drugs. The values of *τ* give an indication on the molecular mobility within the amorphous state, which has implications on its physical and chemical stability. Despite numerous studies in this area, a direct linear relationship between the relaxation time and the actually observed stabilities has not yet been established, suggesting involvement of further factors on amorphous stability [17,35,36,37]. It also has to be remembered that the physico-chemical properties of the amorphous state largely depend on their preparation and handling procedure [38,39]. Additionally, the equations that are used show limitations and therefore cannot precisely describe the mobility behaviour of the amorphous state.

### 2.3. Involvement of S_conf_ in solubility prediction

To predict the maximum solubility advantage of the amorphous state over the crystalline state, *S*_conf_ was determined at the biorelevant temperature of 310 K (37 °C). For the drugs fenofibrate, ibuprofen, tolbutamide, acetaminophen, salsalate and simvastatin the values of *S*_conf_ were calculated from the extrapolated configurational heat capacity data at 310 K as this temperature lies above their respective glass transition temperatures.

However, for the compounds cefuroxime axetil, DRUG A, DRUG B, griseofulvin, troglitazone, lacidipine, indomethacin and nifedipine, the temperature of 310 K lies below the *T_g_*, and these systems exist in the glassy state, hence in a non-equilibrium state, at this temperature. The implication of this is, that thermodynamics should not be applied to these systems as they are not in equilibrium. However, most of the drugs that will be considered for amorphous formulation in the industry will have glass transition temperatures higher than room temperature for physical stability reasons. In order to provide the scientist with an estimation of the solubility increase, the *S*_conf_ of the glassy drugs can be determined by extrapolating the *Cp*_conf_ to the temperature region below the *T_g_*. The values for these drugs should, however, only be used with the knowledge that they were obtained from non-equilibrium conditions.

**Table 2:** Parameters required for the calculation of *τ* using the AG equation (*^a^* relaxation time at *T_g_* − 20 K).

	Δ*H*(*T_g_*)	*T*_0_	*T_f_*	*τ^a^*
Drug	kJ mol^−1^	K	K	h
Cefuroxime axetil	391	256	352	0.4
DRUG B	460	268	344	1.4
Lacidipine	335	228	320	1.5
Indomethacin	387	239	316	1.5
Tolbutamide	265	189	275	3.9
Nifedipine	480	254	314	4.4
Fenofibrate	357	198	249	6.7
Acetaminophen	385	227	291	7.8
Salsalate	236	179	278	8.9
DRUG A	510	271	333	17.8
Griseofulvin	568	292	359	18.9
Troglitazone	533	272	331	19.2
Simvastatin	412	236	300	19.2
Ibuprofen	258	267	223	23.6

In Table 3 the results for the drugs are presented. It can clearly be seen that at a temperature of 310 K these 14 drugs already showed considerable differences in the values for the configurational entropy and hence the solubility ratios.

It can be shown that the theoretical solubility improvement may be as high as 435 fold for DRUG A. Also, the extent of theoretical solubility improvement varies considerably with the drugs: Ibuprofen showed an improvement of 3 fold, whereas griseofulvin exhibited a 324 fold increase.

Bearing in mind that the actual observable solubility increase will be much lower, it can be seen that the amorphous state of the drug shows favourable solubility properties compared to its crystalline counterpart.

## 3. Experimental Section

### 3.1. Materials

These studies used 14 drugs. Study compounds used in this work were of analytical grade and used as received from the respective suppliers. Acetaminophen, fenofibrate ibuprofen, indomethacin, nifedipine, salsalate and tolbutamide were purchased from Sigma Chemicals (Dorset, UK). Cefuroxime axetil, griseofulvin, lacidipine, simvastatin and troglitazone were provided by GlaxoSmithKline (Harlow, UK). DRUG A and DRUG B were synthesized by GSK R&D.

**Table 3:** *S*_conf_, *G*_conf_ and calculated solubility ratio between amorphous and crystalline drugs (* values obtained from non-equilibrium conditions).

	*S*_conf_	*G*_conf_	*σ^a^*/*σ^c^*
Drug	J mol^−1^ K^−1^	J mol^−1^ K^−1^	
Cefuroxime axetil*	84	6.38	12
DRUG A*	129	15.66	435
Griseofulvin*	83	14.90	324
DRUG B*	81	11.80	98
Troglitazone*	63	7.34	17
Lacidipine*	34	12.70	138
Fenofibrate	80	4.01	5
Ibuprofen	72	2.49	3
Indomethacin	40	11.99	105
Nifedipine	38	11.36	82
Tolbutaminde	45	5.07	7
Acetaminophen	22	8.79	30
Salsalate	52	5.41	8
Simvastatin	38	8.50	27

### 3.2. Equipment

A TA Instruments DSC Q100 (TA Instruments - Waters LLC, New Castle, USA) equipped with an RCS cooling system was used. The instrument was calibrated in the modulated mode for heat capacity using sapphire. A modified procedure of calibration was used according to Hill *et al.* [40]. The suitability of the calibration and the experimental conditions were checked by comparison to the literature values of dry crystalline sucrose [41]. A sample size of 3–5 mg was prepared and lightly crimped into aluminium sample pans (TA Instruments). The heating rate and modulation parameters were compound dependent and the parameters were chosen in order to ensure the separation of the non-reversing thermal events from the reversing events.

### 3.3. Thermodynamic involvement of S_conf_

#### 3.3.1. MTDSC analysis of the configurational thermodynamic properties

All samples were prepared in situ in the DSC instrument, in order to minimize heat transfer effects due to weight differences, geometry of the sample or packing in the DSC pan. The same sample was used to determine the heat capacity in the crystalline and the amorphous state. Configurational heat capacity measurements were carried out at a heating rate of 1 K min^−1^ using an amplitude of ±0.5 K and a modulation period of 100 s. The reversing heat capacity was obtained by deconvoluting the total heat capacity using TA Universal Analysis 2000 Software. After measurement of the crystalline heat capacity, the same sample was melted, quench cooled in the DSC and a subsequent run was carried out to determine the amorphous properties. All measurements were carried out in triplicate.

Calculations of the configurational free energy, enthalpy and entropy were carried out using Matlab (R2008a, The MathWorks Inc., Natick, MA, USA). The Matlab script together with detailed comments is available as supplementary information to this article together with a sample dataset of the drug lacidipine.

#### 3.3.2. Physical stability above the glass transition temperature

Amorphous samples were prepared within the DSC instrument by heating approximately 5 mg of each drug to its melting point and then cooling the sample to 253 K at a cooling rate of 20 K min^−1^. Based on the method by Zhou *et al.* [33], drugs were heated through their *T_g_* and the order of stability was assessed by relating the recrystallisation temperature to the glass transition and melting temperature via the a reduced recrystallisation temperature scale (*T_c_* − *T_g_*)/(*T_m_* − *T_g_*).

This transformation represents a normalised measure and provides a means of comparing drugs with different glass transition temperatures. Drugs that did not recrystallise under these conditions were regarded as stable.

### 3.4. Kinetic involvement of S_conf_

Calculation of the relaxation time using the Adam-Gibbs equation is achieved through a number of successive steps. These steps will briefly be mentioned in this section, however, an in depth explanation of the equations can be found elsewhere [19,42,43,44].

1. Measuring the glass transition event in a DSC at different heating rates results in different values for the temperature of the glass transition. The *T_g_* is a kinetic event and therefore dependent on heating rate, following an Arrhenius equation. This can be used in order to calculate the activation enthalpy of the glass transition using the following equation:
  (14) $$\frac{\Delta H(T_{g})*}{R}=\frac{d\ln q}{d(1/T_{g})}$$
  with the heating rate *q* in K min^−1^.
  The heating rate dependence of the glass transition was measured at heating rates of 1, 2, 5, 10 and 20 min^−1^ in conventional DSC mode. The glass transition temperature was taken as the inflection point of the step change. Samples were melted in the DSC and subsequently cooled to 253 K. Fenofibrate and ibuprofen were cooled to 213 K. The samples were equilibrated at a starting temperature 50 K below *T_g_*. The drugs were then heated through the *T_g_* at a given heating rate to 20 K above their *T_g_*. They were cooled back to the starting temperature at the same rate and then heated once more. Measurements were taken from the second heating run. All measurements were performed in triplicate.
2. After plotting ln *q* vs. 1/*T_g_* for all 14 drugs, the value of the activation enthalpy at *T_g_* (Δ*H*(*T_g_*)) was determined by multiplying the slope with the gas constant *R*. An example is shown in Figure 7. The heating rate dependence of simvastatin is presented here.

**Figure 7:**
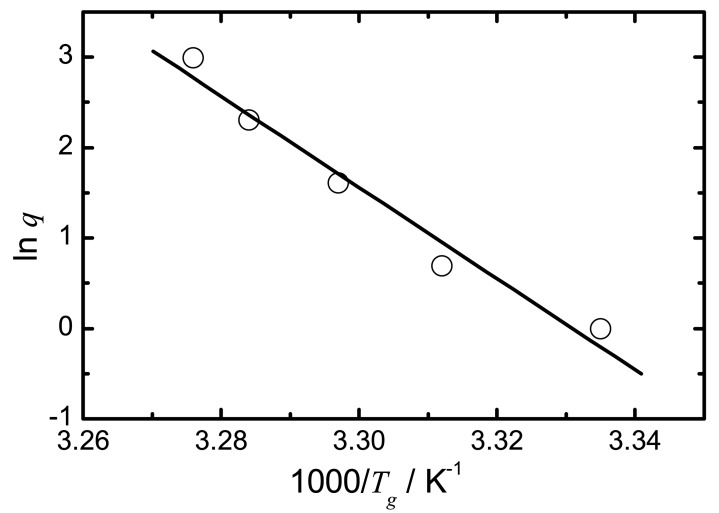
Arrhenius plot of *lnq* vs. 1/*T_g_* to obtain the activation enthalpy at *T_g_* for simvastatin (*r*^2^ = 0.963).
3. With knowledge of the activation enthalpy, the parameters *m* and following that value, *D* can be calculated using the following two equations:
  (15) $$m=\frac{d\log\tau }{d(T_{g}/T)}=\frac{\Delta H(T_{g})*}{(\ln10)RT_{g}}$$
  (16) $$D=\frac{\ln10m_{\min}^{2}}{m-m_{\min}}$$
  where *D* is Angell's strength parameter, *m* is the fragility index and *m*_min_ the minimum fragility value = 16.
4. In the AG equation the temperature of no structural mobility, *T*_0_, is used. Theoretically this temperature is similar to the Kauzmann temperature and can be calculated using:
  (17) $$T_{0}=T_{g}\left(1-\frac{m_{\min}}{m}\right)$$
  where *T*_0_ is the temperature of no structural mobility in Kelvin.
5. The last unknown parameter of the AG equation is the fictive temperature, which includes the time dependent component of the configurational entropy. The fictive temperature can be estimated from Equation 18 by using the heat capacities of the glass, the super-cooled liquid and the crystal.
  (18) $$\frac{1}{T_{f}}=\frac{\gamma_{Cp}}{T_{g}}+\frac{1-\gamma_{Cp}}{T},\;\text{where}$$
  (19) $$\gamma_{Cp}=\frac{Cp^{l}-Cp^{g}}{Cp^{l}}-Cp^{x}$$
  with *T_f_* fictive temperature in Kelvin, *γ_Cp_* the ratio of heat capacities of amorphous and crystalline state and *Cp^l^*, *Cp^g^*, *Cp^x^* the heat capacities of the super-cooled liquid, glass and crystalline state respectively.

This 5 step procedure resulted in the ability to use the AG equation for the calculation of the relaxation time at any temperature. As an example, the temperature 20 K below the respective *T_g_* was chosen. This ensured that all drugs were compared under the same conditions, as the glass transition temperatures for the drugs span values from 363 to 229 K.

## 4. Conclusions

This article has highlighted the importance of the involvement of the configurational entropy for the amorphous state. We have shown that through simple DSC measurements and by developing a very simple Matlab programme, *S*_conf_ can be rapidly calculated at any given temperature and as a result from this, a variety of information regarding the thermodynamic behaviour, molecular mobility and solubility predictions can be obtained.

## Supplementary Information

In the electronic supplementary information we have included the Matlab script s_conf.m used to calculate the *H*_conf_, *S*_conf_ and *G*_conf_ as outlined in this paper. In order to run the script the file s_conf.m either needs to be in the same directory as the DSC data or it needs to be added to the Matlab path (see Matlab documentation).

### Input

The experimental DSC data needs to be in a plain ASCII file which contains two columns of data. the first column is the temperature in °C, the second column is the heat capacity in J g^−1^ K^−1^. This data was generated as described in section Together with this data file a second file lacidipine_powder.txt needs to be present in the same directory which contains additional data about the sample:
sample name: lacidipine powderM_w (g/mol): 455.54H_m (Jmol – 1): 49152.77S_m (Jmol – 1K – 1): 107.90T_g (K): 325.23T_0 (K): 200T_end_l (K): 310T_start_h (K): 330T_m (K): 455.57

Both example files are provided in the electronic supplement.

In order to process the data the script is called using the following syntax:

s_conf(‘lacidipine_powder.csv’,20)

In this example only the specified file is processed. If multiple files are to be analysed at the same time all files should be placed in the same folder and the following command can be used: s_conf(‘*.csv’). For further options use the command help s_conf at the Matlab prompt.

### Output

The script is written such that it will sum up the experimental data points and only use the data from the linear fit once the range of the experimental data points is exceeded.

**Figure 8:**
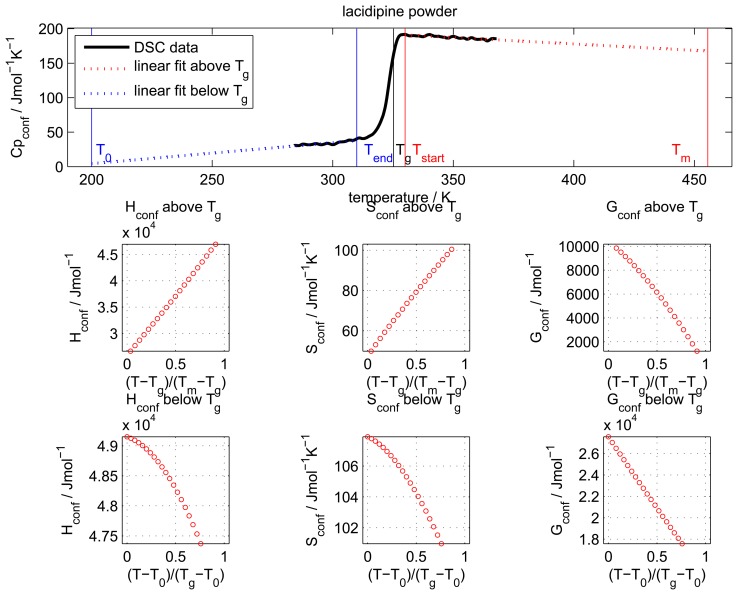
Output figure of the Matlab script showing the experimental DSC trace, the data provided by the user for *T_g_*, *T_m_* and the boundaries for the data fits (top row) together with the *H*_conf_, *S*_conf_ and *G*_conf_ above (middle row) and below *T_g_* (bottom row).

Upon running the script Matlab produces two outputs: a figure showing the data together with the linear fits and a graphical representation of the results (Figure 8, default output format is .eps) and an ASCII text file containing the results in a numerical output.

The ASCII file starts with a summary of the information provided by the user:
########################################################################### CALCULATION OF THE CONFIGURATIONAL ENTHALPY AND ENTROPY ###########################################################################*****DATA PROVIDED BY THE USER***************************************49152.77,Enthalpy of fusion H_m (Jmol–1)107.90,Entropy of fusion S_m (Jmol–1)325.23,Glass transition temperature T_g (K)200.00,T_0, start temperature for the linear fit below T_g (K)310.00,Final temperature for the linear fit below T_g (K)330.00,Start temperature for the linear fit above T_g (K)455.57,Melting point T_m (K)

It then provides the fitting parameters for the fit lines above and below the *T_g_*:
*****FITTING PARAMETERS ABOVE T_g************************************−0.18653,Linear fit parameter a (y = ax + b)0.00099,Standard deviation of a252.49942,Linear fit parameter b (y = ax + b)0.34665,Standard deviation of b0.80405,Linear fit parameter Rˆ21.00946,Linear fit parameter RMSE*****FITTING PARAMETERS BELOW T_g*************************************0.30380,Linear fit parameter a (y = ax + b)0.00204,Standard deviation of a−56.43066,Linear fit parameter b (y = ax + b)0.60560,Standard deviation of b0.79127,Linear fit parameter Rˆ21.16568,Linear fit parameter RMSE

After this section the results for *H*_conf_, *S*_conf_ and *G*_conf_ above and below *T_g_* are provided:
*****CALCULATION RESULTS ABOVE T_g************************************T (K),(T–T_g)/(T_m–T_g),H_c (Jmol–1),S_c (Jmol–1K–1),G_c (Jmol–1)329.99,0.0365,26644.190,49.895,10179.245335.99,0.0826,27688.364,53.085,9852.354341.99,0.1286,28732.506,56.214,9507.906347.99,0.1746,29765.350,59.252,9146.291353.99,0.2207,30793.177,62.220,8768.063359.99,0.2667,31811.830,65.108,8373.656365.99,0.3127,32819.692,67.915,7963.552371.99,0.3588,33859.732,70.760,7537.766377.99,0.4048,34914.097,73.594,7096.127383.99,0.4508,35975.179,76.399,6638.829389.99,0.4969,37042.978,79.174,6165.946395.99,0.5429,38117.494,81.921,5677.543401.99,0.5889,39197.954,84.640,5173.685407.99,0.6350,40285.900,87.334,4654.423413.99,0.6810,41380.564,90.004,4119.814419.99,0.7270,42481.950,92.650,3569.903425.99,0.7731,43589.407,95.271,3004.747431.99,0.8191,44704.578,97.873,2424.383437.99,0.8651,45826.674,100.454,1828.842443.99,0.9112,46955.696,103.015,1218.151
*****CALCULATION RESULTS BELOW T_g****************************************************************T (K),(T_g–T_0)/(T_g–T_0),H_c (Jmol–1),S_c (Jmol–1K–1),G_c (Jmol–1),H (Jmol–1),S (Jmol–1K–1)200.01,0.0001,49152.696,107.900,27571.691,0.074,0.000205.01,0.0400,49127.229,107.774,27032.492,25.541,0.126210.01,0.0799,49094.170,107.615,26494.006,58.600,0.285215.01,0.1199,49053.517,107.423,25956.397,99.253,0.477220.01,0.1598,49005.271,107.202,25419.821,147.499,0.698225.01,0.1997,48949.381,106.951,24884.427,203.389,0.949230.01,0.2396,48885.943,106.672,24350.358,266.827,1.228235.01,0.2796,48814.911,106.366,23817.751,337.859,1.534240.01,0.3195,48736.286,106.035,23286.734,416.484,1.865245.01,0.3594,48650.068,105.680,22757.435,502.702,2.220250.01,0.3993,48556.257,105.301,22229.972,596.513,2.599255.01,0.4393,48454.853,104.899,21704.461,697.917,3.001260.01,0.4792,48345.759,104.476,21181.012,807.011,3.424265.01,0.5191,48229.162,104.032,20659.733,923.608,3.868270.01,0.5591,48104.972,103.567,20140.725,1047.798,4.333275.01,0.5990,47973.189,103.084,19624.086,1179.581,4.816280.01,0.6389,47833.813,102.582,19109.913,1318.957,5.318285.01,0.6788,47686.827,102.061,18598.296,1465.943,5.839290.01,0.7188,47532.153,101.523,18089.324,1620.617,6.377295.01,0.7587,47369.912,100.969,17583.083,1782.858,6.931

The remainder of the file provides the data points used to plot the linear fits above (column 1 and 2) and below (column 3 and 4) the *T_g_*:
*****LINEAR FIT DATAPOINTS****************************************************************T (K),fitted cp (Jmol–1K–1),T (K),fitted cp (Jmol–1K–1)330.0019,190.9434,200.0000,4.3300330.0069,190.9424,200.0043,4.3313330.0102,190.9418,200.0086,4.3326330.0152,190.9409,200.0128,4.3339330.0202,190.9399,200.0171,4.3352330.0236,190.9393,200.0214,4.3365330.0286,190.9384,200.0257,4.3378330.0336,190.9375,200.0300,4.3391330.0369,190.9368,200.0342,4.3404330.0402,190.9362,200.0385,4.3417330.0452,190.9353,200.0428,4.3430330.0502,190.9344,200.0471,4.3443…

## Supplementary Files

Supplementary File 1 RAR-Document (RAR, 380 KB)
